# Point mutations in the tumor suppressor Smad4/DPC4 enhance its phosphorylation by GSK3 and reversibly inactivate TGF-β signaling

**DOI:** 10.1080/23723556.2015.1025181

**Published:** 2015-04-14

**Authors:** Hadrien Demagny, Edward M De Robertis

**Affiliations:** Howard Hughes Medical Institute and Department of Biological Chemistry; University of California; Los Angeles, CA USA

**Keywords:** BMP, CDK, Caco-2, DPC4, GSK3, MAPK, pancreatic cancer, prostate cancer, β-TrCP, Wnt/STOP

## Abstract

The tumor suppressor Smad4/DPC4 is an essential transcription factor in the TGF-β pathway and is frequently mutated or deleted in prostate, colorectal, and pancreatic carcinomas. We recently discovered that Smad4 activity and stability are regulated by the FGF/EGF and Wnt signaling pathways through a series of MAPK and GSK3 phosphorylation sites located in its linker region. In the present study, we report that loss-of-function associated with 2 point mutations commonly found in colorectal and pancreatic cancers results from enhanced Smad4 phosphorylation by GSK3, generating a phosphodegron that leads to subsequent β-TrCP–mediated polyubiquitination and proteasomal degradation. Using chemical GSK3 inhibitors, we show that Smad4 point mutant proteins can be stabilized and TGF-β signaling restored in cancer cells harboring such mutations.

## Abbreviations

Bone Morphogenetic Protein: (BMP)
Cyclin-dependent kinase: (CDK)
Caco-2: cell line
Deleted in prancreatic carcinoma 4: (DPC4)
Glycogen Synthase Kinase 3: (GSK3)
Mitogen-Activated Protein Kinase: (MAPK)
pancreatic: cancer
prostate: cancer
beta-Transducing Repeat-containing protein: (beta-TrCP)
Wnt-induced STabilization Of Proteins: (Wnt/STOP).

## Introduction

The transforming growth factor-β (TGF-β) family of cytokines constitutes the largest group of growth factors in humans and regulates many aspects of cell behavior such as differentiation, migration, and apoptosis.^1^ The TGF-β pathway exerts potent antitumor effects and its misregulation often results in cancer progression.^2^ In the canonical TGF-β signaling pathway, activated TGF-β receptors phosphorylate C-terminal serine residues of the transcription factors Smad1/5/8 for bone morphogenetic proteins (BMPs) or Smad2/3 for the TGF-β/activin branch of the pathway.^3^ The transcription factor Smad4, also called deleted in pancreatic carcinoma 4 (DPC4), functions as a co-Smad that binds to receptor-phosphorylated Smads (R-Smads) and is an essential downstream determinant in TGF-β signaling that was, until recently, considered a constitutive component of the pathway.^3^

Although receptor-phosphorylated Smads or TGF-β receptors can be mutated or deleted in some cancers,^4^ Smad4 appears to be the main barrier to tumor progression and is frequently mutated, with devastating effects, in prostate and pancreatic carcinomas.^5^ Mutations in Smad4/DPC4 have been identified in approximately 50% of pancreatic adenocarcinomas,^6,7^ suggesting a pivotal role during pancreatic cancer progression. Tumors lacking functional Smad4 are more proliferative, invasive, and angiogenic, and consequently more likely to form metastases.^8^ Among patients undergoing surgical removal of pancreatic adenocarcinoma, survival was found to be significantly longer for patients whose tumors express Smad4 protein.^8^

The ubiquitin proteasome pathway regulates degradation of most proteins in mammalian cells, and E3 ubiquitin protein ligases (E3-ligases) determine the specificity and timing of the ubiquitination process for given substrates.^9^ Skp1-Cullin1-F-box (SCF) E3 ligases recognize specific protein substrates through their variable F-box proteins.^10^ β-transducin repeat-containing protein (β-TrCP), a member of the Fbw subfamily of F-box proteins, is known to recognize various phosphorylated substrates, including β-catenin,^11^ IkappaB,^11,12^ nuclear factor (NF)-kappaB B2,^13^ NF-kappaB p105,^14^ Snail,^15^ and TAZ.^16,17^ Interestingly, several of these proteins are substrates for glycogen synthase kinase-3 (GSK3), a Wnt- and phosphoinositide 3-kinase (PI3K)-regulated kinase that recognizes pre-phosphorylated substrates. For phosphorylation of a Serine/Threonine (Ser/Thr) residue, GSK3 prefers a phospho-Ser/Thr at the n + 4 position, which is known as the priming phosphorylation site.^18^

We recently reported that Smad4 activity and stability are directly regulated through GSK3 phosphorylations in the linker region of Smad4 that are primed by the mitogen-activated protein kinase (MAPK) extracellular signal-regulated kinase (Erk).^19^ We found that fibroblast growth factor (FGF)/epidermal growth factor (EGF) stimulation leads to Smad4 phosphorylation by Erk of the canonical MAPK site (PXTP) located at Threonine 277. Phosphorylation of this site recruits transcriptional co-activators, most likely p300, allowing Smad4 to reach peak transcriptional activity.^19,20^ However, MAPK also primes Smad4 for 3 subsequent GSK3-mediated phosphorylations that cause transcriptional inhibition and generate a phosphodegron that is used as a docking site by β-TrCP, leading to protein polyubiquitination and proteasomal degradation.^19^ Wnt signaling inhibits GSK3 activity^22,23^ and therefore potentiates Smad4 activity, particularly at low concentrations of TGF-β.^19^ Thus, the linker domain of Smad4 contains a growth factor-regulated transcription activation domain that integrates Wnt, FGF/EGF, and TGF-β signaling. This convergence of signaling pathways via Smad4 plays an important role during early Xenopus development, where it regulates cell competence to mesoderm induction on the dorsal side of the embryo.^19^

Interestingly, Wan et al. reported that point mutations commonly found in colorectal and pancreatic carcinomas enhance Smad4 degradation by increasing β-TrCP–mediated Smad4 polyubiquitination.^24,25^ Our finding that β-TrCP binding to Smad4 is regulated by GSK3 phosphorylations raised the question of whether this increased binding between β-TrCP and Smad4 resulted from an increase in Smad4 GSK3 phosphorylations. In the present study, we report that Smad4 proteins harboring 2 point mutations commonly found in human pancreatic and colorectal cancers (Pro130Ser and Asn351His) have enhanced phosphorylation by GSK3 and are highly unstable. GSK3 inhibitors restored Smad4 stability and TGF-β signaling in smad4−/− mammary tumor cells expressing Smad4 mutant proteins. In addition, in the colon carcinoma Caco-2 cell line, which endogenously expresses the Smad4N351H mutation and is refractory to TGF-β treatment, TGF-β signaling can be restored by simply treating cells with the GSK3 inhibitor lithium chloride (LiCl). In conclusion, the present findings reveal that some mutations found in human cancers do not alter Smad4 function but rather inhibit TGF-β signaling by enhancing GSK3 phosphorylation of Smad4, which leads to its subsequent degradation. These mutant proteins have retained their ability to transduce the TGF-β signal, provided that their instability is reversed by inhibiting GSK3 activity.

## Results

### Smad4 Pro130Ser and Asn351His mutants have enhanced GSK3 phosphorylation

**Figure 1.**
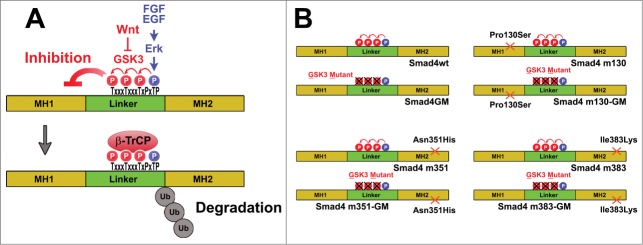
Regulation of Smad4 activity and stability by linker phosphorylations, and mutations used in this study. (**A**) FGF or EGF stimulate Smad4 phosphorylation by Erk at Thr 277, which has a dual function. First, it allows Smad4 to reach peak transcriptional activity by recruiting transcriptional co-activators.^18^ Second, MAPK/Erk phosphorylation primes Smad4 for GSK3 phosphorylations that cause transcriptional inhibition and also generate a phosphodegron that serves as a docking site for the ubiquitin E3 ligase β-TrCP. Thus, both the activity and the stability of Smad4 are regulated by the FGF/EGF and Wnt signaling pathways. Diagram modified from ref. 19. (**B**) Diagrams of Smad4 constructs encoding Smad4 wild-type (Smad4-wt) or point mutations commonly found in human cancers: Pro130Ser; Asn351His and Ile383Lys (referred to as Smad4m130, Smad4m351, and Smad4m383, respectively) with intact linker GSK3 phosphorylation sites or phosphorylation-resistant mutants (Thr to Val) for the GSK3 sites (Smad4-GM)

We previously showed that Smad4 stability is controlled by Wnt-regulated GSK3 phosphorylations that generate a docking site for the ubiquitin E3 ligase β-TrCP.^19^ Others had observed that point mutations commonly found in colorectal and pancreatic cancers enhance the interaction between Smad4 and β-TrCP.^25^ Our discovery that the binding of Smad4 to β-TrCP requires GSK3 phosphorylation suggested that Smad4 containing point mutations may have an increased susceptibility to GSK3 phosphorylation. This, in turn, would generate a phosphodegron recognized by β-TrCP, triggering polyubiquitination and degradation in the proteasome (**Fig. 1A**).

To test this hypothesis, we constructed a series of Smad4 mutants harboring 3 point mutations in the MH1 or MH2 domain that are commonly found in human cancers (Fig. 1B).^4^ Two of these, Pro130Ser and Asn351His (referred to herein as Smad4m130 and Smad4m351), had been reported to be rapidly degraded as a consequence of increased binding to β-TrCP.^25^ On the other hand, binding between Smad4 Ile383Lys (Smad4m383) and β-TrCP was not found to be significantly enhanced,^25^ and was used as a control. To test the role of the newly discovered GSK3 linker phosphorylation sites in the degradation of Smad4 mutants, we generated these point mutations with either intact linker GSK3 phosphorylation sites or mutated (Thr to Val) GSK3 phosphorylation sites (designated as Smad4m130-GM; Smad4m351-GM and Smad4m383-GM) (Fig. 1B).

**Figure 2.**
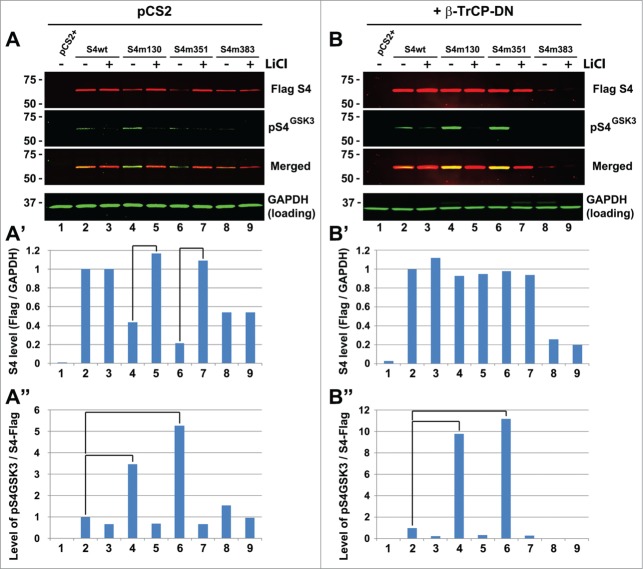
Enhanced phosphorylation of Smad4 Pro130Ser and Smad4 Asn351His. (**A**) Western blot analysis showing that Smad4 proteins harboring point mutations are less stable than their wild-type counterparts. Mutations Pro130Ser and Asn351His increase phosphorylation by GSK3 marked by a custom-made pSmad4^GSK3^ antibody. Cells were preincubated with LiCl for 5 h. The gel shown is a representative experiment that was replicated in 2 independent experiments. (**A**’) Quantification of protein stability, normalized for GAPDH levels, from the western blot above. (**A**”) Quantification of protein phosphorylation by GSK3 (normalized for total Flag-Smad4 protein in each lane) of the same western blot. Note that LiCl treatment blocks GSK3 phosphorylation while increasing protein stability. (**B**) Inhibition of β-TrCP using transfected DN-β-TrCP showing that Smad4m130 and m351 are more highly phosphorylated than wild-type Smad4 and that their rapid degradation requires β-TrCP. This experiment was independently replicated twice. **(B’)** Quantification of Flag-Smad4 protein (normalized for GAPDH) from western blot above. **(B”)** Quantification of phospho-Smad4^GSK3^, normalized for total Flag-Smad4, from the same western blot. Note that in these experiments FGF addition was not required because 10% serum provided a basal level of MAPK activation.

When these Smad4 constructs were transfected into human embryonic kidney cells (HEK-293) we found that all 3 mutants were less stable than the Smad4 wild-type (wt) protein (Fig. 2A and A', compare lanes 2, 4, 6, and 8).^25,26^ To determine the level of GSK3 phosphorylation of Smad4 mutant proteins, the western blot was also immunostained with an affinity-purified phospho-specific antibody raised against the 2 first Smad4 linker GSK3 phosphorylation sites (phospho-Thr 273 and 269, referred to as pS4GSK3 Ab in Fig. 2A).^19^ Because the expression levels of Smad4wt and its mutants were different, the intensity of the pS4GSK3 bands was quantitated using the Li-Cor infrared imaging system and normalized over the total level of Flag-tagged Smad4 in each lane (pS4GSK3/S4-Flag, Fig. 2A”). This normalization showed that Smad4m130 and Smadm351 were more highly phosphorylated by GSK3 compared to the wild-type protein (Fig. 2A”, compare lane 2 to lanes 4 and 6, brackets). The phosphorylations were inhibited by the GSK3 inhibit or lithium chloride (LiCl) and therefore required GSK3 activity (Fig. 2A). Phosphorylation of Smad4m383 (used here as a control) was not significantly different from that of the wild-type protein (Fig. 2A”, bars 2 and 8).

The enhanced GSK3 phosphorylation caused by Smad4m130 and m351 mutations became more prominent when their rapid degradation was inhibited by co-transfection of a dominant-negative form of β-TrCP (DN-β-TrCP lacking the F-box domain, which causes stabilization of pSmad4GSK3,^19,27^) (Fig. 2B, note the yellow signal in the Merged panel and brackets in Fig. 2B”). We had previously shown that transfection with DN-β-TrCP inhibits the rapid degradation of pSmad4GSK3 compared to untransfected cells.^19^

Importantly, treatment of cells with the GSK3 inhibitor LiCl restored the stability of Smad4m130 and Smad4m351 to levels similar to those of Smad4wt (Fig. 2A’, brackets). When Smad4 constructs were co-transfected with DN-β-TrCP, the levels of Smad4m130 and Smad4m351 were not significantly different from those of Smad4wt and were no longer affected by LiCl treatment (Fig. 2B’). Taken together, these results indicate that Smad4m130 and Smad4m351, but not Smad4m383, are more susceptible to GSK3 phosphorylation and consequently less stable. Chemical inhibition of GSK3 restores the stability of these mutants by inhibiting their phosphorylation by GSK3 and subsequent β-TrCP degradation.

### Inhibition of GSK3 activity restores TGF-β signaling in cells expressing Smad4m130 and Smad4m351

**Figure 3.**
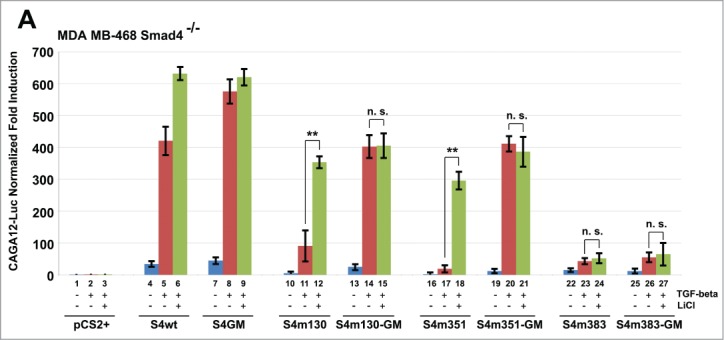
Inhibition of GSK3 activity restores TGF-β signaling in cells expressing Smad4m130 and Smad4m351. MDA-MB-468 mammary carcinoma cells (*smad4*^−/−^) were transfected with equal amounts of various DNAs and treated as indicated with TGF-β1 (1 ng/mL) or the GSK3 inhibitor LiCl (30 mM). Note that Smad4m130 and m351 are less active than Smad4wt. Importantly, TGF-β signaling can be restored in these cells by LiCl treatment or by mutating the GSK3 phosphorylation sites (GM-mutants, Thr to Val). The Smad4m383 construct does not respond to GSK3 inhibition, presumably because the mutation inactivates the function of the transcription factor. It is remarkable that 2 almost complete loss-of-function mutations can be reversed by simply adding a GSK3 inhibitor.

A central question in this investigation was whether Smad4m130 and Smad4m351 retained their ability to transduce the TGF-β signal when their β-TrCP-mediated degradation was blocked by GSK3 inhibitors. To investigate this, we used mammary carcinoma MDA MB-468 cells that do not express endogenous Smad4 because of a deletion of the Smad4 gene and are therefore unresponsive to TGF-β.^21^ Cells were transfected with the TGF-β-specific reporter CAGA12-luciferase,^28^ and dual luciferase reporter gene assay experiments were carried out (**Fig. 3**).

**Figure 4.**
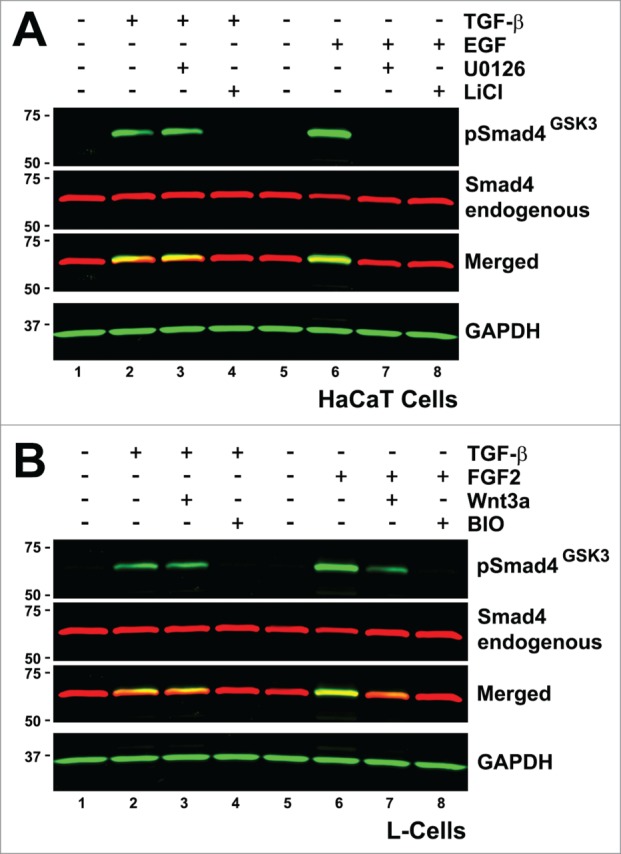
GSK3 phosphorylation can be induced by MAPK/Erk activation, or by TGF-β via an Erk- and Wnt-independent mechanism. (**A**) Western blot showing that GSK3 phosphorylation of the Smad4 linker region can be induced by EGF through an Erk-dependent mechanism or by TGF-β through a different Erk-independent mechanism. These results show that TGF-β can induce linker phosphorylation of Smad4 but that in this case Erk is not the priming kinase. (**B**) GSK3 linker phosphorylations of Smad4 were inhibited by Wnt3a when induced by FGF, whereas the TGF-β induced linker phosphorylations were unaffected by Wnt3a.

Co-transfection of Smad4-wt into these smad4−/− cells restored TGF-β responsiveness, which was moderately increased by LiCl (Fig. 3, bars 1–6). In contrast, cells expressing Smad4m130 and Smad4m351 transduced the TGF-β signal very poorly but their signaling levels were dramatically increased by the addition of LiCl (Fig. 3, compare bars 11 to 12 and 17 to 18). Importantly, when cells were transfected with their counterpart GSK3-resistant forms (Smad4m130-GM and Smad4m351-GM, respectively), TGF-β caused a strong response but the potentiation by LiCl was lost (Fig. 3, bars 13–15 and 19–21). Since replacing Smad4m130 and Smad4m351with their GSK3-insensitive mutants eliminated LiCl potentiation, we conclude that the observed potentiation of TGF-β by LiCl is mediated by the GSK3 phosphorylation sites of Smad4 and not by other components of the signal transduction pathway. In contrast, the low activity of Smad4m383 was not reactivated by LiCl or by mutation of its GSK3 sites (Fig. 4, bars 22–27) indicating that the Ile383Lys mutation has some functional defect in transducing the TGF-β signal that cannot be reversed by GSK3 inhibition.

We conclude that the GSK3 phosphorylation sites of Smad4 mediate the loss-of-function of the Pro130Ser and Asn351His Smad4 mutations by promoting their degradation via linker phosphorylations. Remarkably, GSK3 inhibitors can restore the transcriptional function of these mutations, suggesting novel therapeutic possibilities.

### TGF-β induced linker phosphorylations of Smad4

In the course of this study we made the unexpected observation that linker phosphorylation of Smad4 could also be triggered by TGF-β stimulation in HaCaT cells (**Fig. 4A**, lane 2). Interestingly, these phosphorylations induced by TGF-β, although completely inhibited by the GSK3 inhibitor LiCl, were unaffected by the MEK inhibitor U0126, suggesting the involvement of a kinase different from Erk in priming of the GSK3 sites (**Fig. 4A**, lanes 3 and 7). A prime candidate would be a kinase belonging to the family of nuclear CDKs associated with transcription such as CDK8/9. Indeed, the linker region of R-Smads was shown to be phosphorylated by nuclear CDKs in response to TGF-β or BMP stimulations.^29,30^ Although this possibility is attractive, whether the Smad4 phosphodegron is also primed by CDK8/9 awaits experimental validation.

The GSK3 linker phosphorylations induced by TGF-β differ from those induced by FGF. Indeed, we found that pSmad4^GSK3^ phosphorylations are greatly inhibited by Wnt3a but only when primed by FGF; when the pSmad4^GSK3^ phosphorylation was triggered by TGF-β, Wnt3a treatment had no effect (**Fig. 4B**, compare lanes 2 to 3 and 6 to 7). This suggests that the 2 signaling pathways use different cellular mechanisms to trigger the phosphorylation of Smad4 by GSK3. One possible explanation for this observation is that the FGF-induced GSK3 phosphorylations of Smad4 take place in the cytoplasm, whereas TGF-β induced GSK3 phosphorylation of Smad4 occurs in the nucleus while Smad4 is engaged in transcription and would be independent of the Wnt-destruction complex. This hypothesis would be consistent with the proposed cycle of activation for R-Smad proteins,^29,30^ and with our previous results showing that the regulation of Smad4 activity by Wnt is entirely dependent on an FGF-triggered MAPK/Erk priming mechanism.^18^ We conclude from the results in **Fig. 4** that a second pathway exists for phosphorylation of Smad4 GSK3 sites that is triggered by TGF-β but not regulated by Wnt, as is the case when primed by FGF/EGF.

### Inhibition of GSK3 activity restores TGF-β signaling in Caco-2 cells expressing endogenous Smad4^N351H^

**Figure 5.**
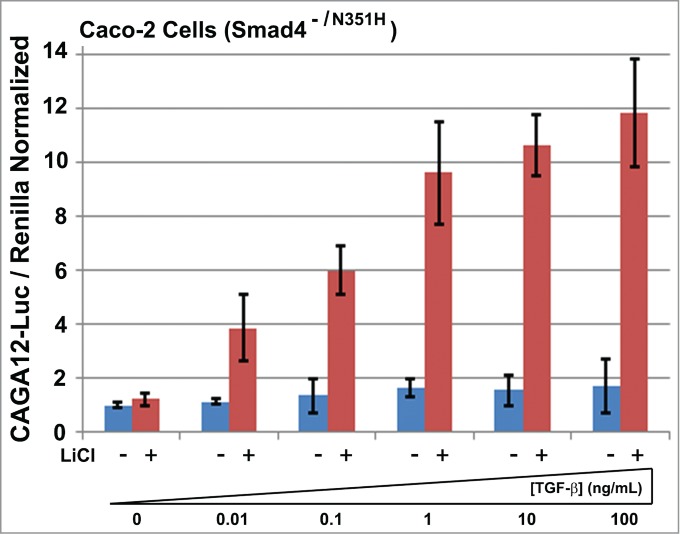
Inhibition of GSK3 restores dose-dependent TGF-β signaling to Caco-2 Smad4^−/N351H^ cells. The GSK3 inhibitor LiCl restores TGF-β responsiveness to the colon carcinoma Caco-2 cell line, which endogenously expresses the Smad4^N351H^ mutant. Cells were treated with 30 mM LiCl for 2 h before addition of the indicated amounts of TGF-β1. Note that Caco-2 cells are unresponsive to TGF-β1, yet in the presence of GSK3 inhibitors dose-dependent signaling is reactivated.

We next investigated whether GSK3 inhibition could restore TGF-β signaling in a human cancer cell line carrying a point mutation in Smad4. We used the colon cancer cell line Caco-2, which has lost one allele of *smad4* while the other allele harbors the Asn351His Smad4 point mutation (*smad4*^−/N315H^).^31^ Caco-2 cells were transfected with the TGF-β–specific reporter CAGA12-luciferase (and pCS2-Renilla for normalization), incubated with or without LiCl, and treated for 8 h with different amounts of TGF-β1 ligand (**Fig. 5**). In the absence of LiCl, Caco-2 cells showed no significant transcriptional response to TGF-β ligand stimulation (**Fig. 5**, blue bars). However, preincubation of the cells with LiCl allowed TGF-β1 to induce the CAGA-12 reporter in a dose-dependent manner (**Fig. 5**, red bars). These results demonstrate that GSK3 chemical inhibition can reactivate TGF-β signaling in cells harboring a point mutation (Asn351His) in the endogenous tumor suppressor Smad4.

## Discussion

**Figure 6.**
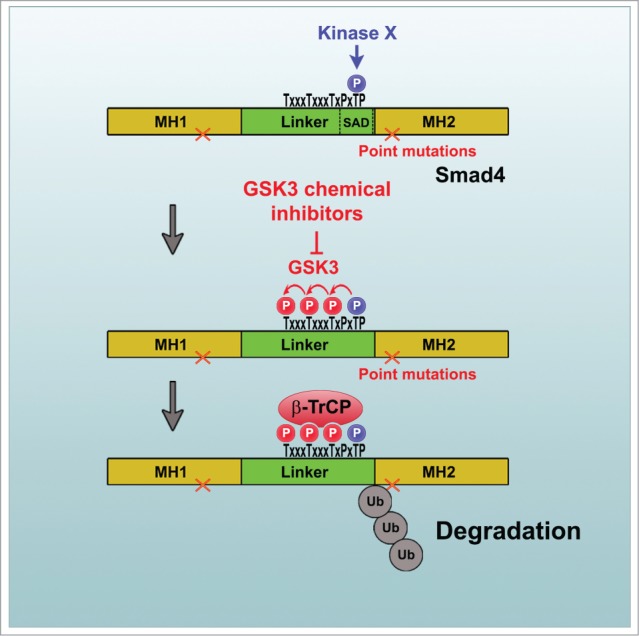
Proposed model of the mechanism by which point mutations in Smad4 increase its degradation via GSK3 phosphorylation. We propose that some Smad4 point mutations commonly found in human cancers may lead to enhanced recruitment of Ras/Erk or other unknown kinases (X kinases) that phosphorylate Thr277, leading to subsequent GSK3 phosphorylations and generating a phosphodegron that is recognized and bound by the E3 ligase β-TrCP. In this model, chemical inhibitors of GSK3 would prevent Smad4 degradation and restore growth control by TGF-β signaling in a subset of Smad4 point mutations.

Smad4 is a critical transcription factor in the TGF-β pathway and loss of Smad4 is associated with malignant progression in prostate, colon, and pancreatic carcinomas.^4,5^ In the present work, we provide evidence that 2 Smad4 missense point mutations found in human cancers (Pro130Ser and Asn351His) increase Smad4 phosphorylation by the GSK3 kinase, thus triggering its degradation (**Fig. 6**). Chemical inhibition of GSK3 blocked the increased phosphorylation caused by these Smad4 mutations and stabilized protein levels in HEK293 cells. Replacing Smad4 with various mutations in a *smad*^−/−^ mammary cancer cell line showed that TGF-β signaling is greatly impaired by point mutations that naturally occur in cancer, and that in 2 of these cases signaling via a synthetic TGF-β reporter gene can be reactivated by simply inhibiting GSK3 activity. In Caco-2 cells, which express Smad4^N351H^ protein, the GSK3 inhibitor LiCl was sufficient to restore TGF-β responsiveness in a cell line that was previously impervious to TGF-β signaling. These findings suggest that the TGF-β intracellular cascade is intact and that the low level of Smad4^N351H^ is the rate-limiting factor in Caco-2 cells. TGF-β signaling can be reactivated provided that Smad4 rapid degradation is blocked by a GSK3 chemical inhibitor in cells that would otherwise behave as TGF-β loss-of-function mutants.

Until recently, Smad4 was believed to function as an unregulated constitutive co-Smad that bound to R-Smads after their activation by C-terminal phosphorylations, causing nuclear translocation of the complex.^1^ However, our recent work has shown that Smad4 is a transcription factor regulated both by FGF/EGF and canonical Wnt signaling.^19^ In the presence of Thr277 phosphorylation by MAPK/Erk, the transcriptional activity of Smad4 is maximal provided that GSK3 activity is inhibited by Wnt. Maximal activity is mediated by the binding of a co-activator, probably p300, to a growth factor-regulated Smad-activating domain (SAD) that includes Thr277.^19,20^ In the absence of Wnt, Thr277 phosphorylation primes 3 GSK3 phosphorylations that generate a phosphodegron recognized by β-TrCP, inhibiting transcription and triggering Smad4 degradation (**Fig. 6**).

Smad4 acts as a barrier to tumor progression because it lies at a signaling crossroads integrating the TGF-β, Ras/Erk, and Wnt signaling pathways.^32^ TGF-β has potent antiproliferative activity through the activation of CDK inhibitors such as p14^Ink4b^ and p21^WAF1^.^33,34^ Many tumors have mutations that activate Ras/Erk and Wnt signaling, causing mitogenic effects that will be counteracted by the increased activity of Smad4 and explaining the barrier effect of this key transcription factor during tumor progression.^5,19^ The ability to reverse Smad4 loss-of-function caused by specific point mutations using pharmacological agents may provide new therapeutic avenues.

Canonical Wnt stabilizes β-catenin by inhibiting its phosphorylation by GSK3.^22,23^ This inhibition requires endocytosis of GSK3 bound to the Wnt receptor complex, initially in LRP6-signalosomes and then in multivesicular bodies (MVBs).^35–37^ GSK3 has many other substrates in addition to β-catenin.^36,38,39^ The Smad4 GSK3 sites first came to our attention in the course of a bioinformatics screen for proteins containing 3 or more GSK3 phosphorylation sites in a row.^19,36^ Many other proteins have been found to be regulated by GSK3 sequestration in membrane organelles after Wnt treatment, in a process that has been designated as Wnt-regulated STabilization Of Proteins, or Wnt/STOP and, as Wnt signaling is maximal during the G2/M phase of the cell cycle, is considered to be essential for protein stabilization in preparation for cell division.^39^ Thus, it is conceivable that the biochemical changes caused by Smad4 point mutations might also apply to other proteins.

The results presented in this study show that some mutations in Smad4 do not alter its activity but rather interfere with the phosphorylation and degradation pathway regulated by Ras/Erk and canonical Wnt/GSK3.^19^ These mutations inactivate Smad4 by enhancing GSK3-mediated phosphorylations that generate a phosphodegron recognized by the E3 ubiquitin ligase β-TrCP, resulting in loss-of-function via protein degradation.^19,25^ It is surprising that mutations in the MH1 and MH2 domains (Pro130Ser and Asn351His, respectively) would similarly affect Smad4 phosphorylations at the distant linker region (**Fig. 6**). One possibility is that these missense mutations might cause misfolding of the protein. Cellular protein kinases may recognize abnormal proteins more readily, generating phosphodegrons that mark them for rapid degradation. When GSK3 is inhibited the protein is no longer marked by phosphorylations, and cellular chaperones may have more time to refold Smad4 into a functional protein. As Wnt/STOP regulation of protein stability affects a multitude of proteins,^39^ it is possible that other, as yet unknown, loss-of-function mutations involved in disease might be alleviated by GSK3 inhibition.

The findings reported here provide new insights into the mechanism by which point mutations in Smad4 cause their rapid degradation in tumor cells. They also suggest potential therapeutic applications in which pharmacological GSK3 inhibitors might stabilize Smad4 and restore TGF-β signaling and growth control in tumors harboring such mutations. Indeed, although E3-ligase inhibitors are poorly characterized as anticancer drugs,^40^ many pharmacological GSK3 inhibitors have been developed. Given the importance of GSK3 in neurodegenerative disorders, inhibitors of varied chemical structure and mechanism of action are the focus of current clinical trials.^41,42^ Future studies will be needed to test the usefulness of GSK3 inhibition *in vivo* in immunodeficient mouse models transplanted with tumors containing Smad4 point mutations; in this regard, Caco-2 cells should provide a good starting point.

## Materials and Methods

### Mammalian cell culture

HEK293 cells (lacking T antigen, which respond very well to TGF-β) were cultured in DMEM supplemented with 10% fetal bovine serum (GIBCO) at 37°C in 5% CO_2_. MDA-MB-468 cells (which lack Smad4) were cultured in DMEM:Ham's-F12 (1:1 vol:vol). Cells were transfected with DNA constructs using BioT (Bioland) 24 h after plating. For all treatments with growth factors, cells were deprived of serum for 18 h and then treated with the indicated concentration of TGF-β1, FGF2 (50 ng/mL), or EGF (10 ng/mL) (R&D Systems). The Erk pathway inhibitor U0126 was used at a concentration of 40 μM. For GSK3 inhibition, cells were treated for the indicated time with the GSK3 inhibitor LiCl used at 30 mM or BIO (Sigma) used at 5 μM.

### Antibodies

The following antibodies were used in this study for the detection of overexpressed proteins: anti-Smad4 monoclonal (Santa Cruz Biotechnology B-8, 1:250), anti-GAPDH (Cell Signaling Technology 14C10, 1:7,000), and anti-pSmad4^GSK3^^18^ used at 1:25,000. Secondary antibodies used were IRDye 800CW donkey anti-rabbit IgG (Li-Cor 926–32213, 1:5,000) and IRDye 680RD donkey anti-mouse IgG (Li-Cor 926–68072, 1:5,000).

### Western blot analyses

For western blot analyses, cells were cultured in 6-well plates, treated as indicated for 4 h, and then lysed in radioimmunoprecipitation assay buffer (RIPA lysis buffer, 0.1% NP40, 20 mM Tris/HCl pH 8, 10% glycerol) supplemented with protease inhibitors (Roche #04693132001) and phosphatase inhibitors (Calbiochem 524629). Western blotting was performed using standard protocols. Odyssey™ Blocking Buffer (LI-COR) diluted in PBS (1:1 ratio) was used to block nitrocellulose membranes for 1 h at room temperature. All antibodies were diluted in PBS:Odyssey™ Blocking Buffer supplemented with 0.1% Tween 20. Blots were incubated with primary antibodies overnight at 4°C. Membranes were then washed extensively with Tris-buffered saline Tween 20 (TBST) and incubated with secondary antibodies for 1 h at room temperature. Images were acquired using an Odyssey 9120 infrared imaging system (LI-COR).

## Plasmid Reagents

Flag-tagged human Smad4 in pCS2+ was previously described.^18^ PCR-based site-directed mutagenesis (QuikChange II Site-Directed Mutagenesis, Stratagene) was employed to generate all Smad4 mutants used in this study. Primers used were as follows: Smad4m130 (GTCTGTGTGAATTCATATCACTACG); Smad4m351 (GTTACTGTTCATGGATACGTG); Smad4m383 (GGTTGCACAAAGGCAAAGGT GTG); Smad4-GM (CATAACAGCGTACCACCTGGGCTGGAAGTAGGGCTGCACC ATAC). Mutations were confirmed by sequencing. HA-tagged human β-TrCP ΔF-box (referred as DN-β-TrCP in **Fig. 2**) was a generous gift from C. Carbone, and was subcloned from pEF61 vector into the expression vector pCS2+.

## Reporter Gene Assays

For luciferase reporter gene assays, HEK293 or MDA-MB-468 cells were transiently transfected in 6-well plates at 80% confluency. The following day, cells were trypsinized and plated on 24-well plates. Cells were allowed to attach to the plastic surface, preincubated with LiCl (30 mM) for 2 h, and treated with the indicated concentrations of TGF-β1 (R&D Systems) for an additional 8 h. Growth factors were added at cell confluency of 60% or less; this is important because high confluency inhibits TGF-β signaling.^18,27^ To normalize the transfection efficiency, pCS2^+^-Renilla was co-transfected. The following amounts of plasmids were used per well: 1.2 μg CAGA12 reporter; 0.4 μg pCS2^+^-Renilla; 0.4 μg pCS2^+^-Smad4 in its wild type and mutant forms. DNA levels in each well were adjusted by addition of empty pCS2^+^ vector so that each well received a total of 2 μg DNA. After treatment, cells were lysed with 180 μL of Passive Lysis Buffer (Promega) and luciferase assays were performed with the Dual-Luciferase Reporter Assay System (Promega) according to the manufacturer's instructions using a Glomax Luminometer (Promega).

## Statistical Analyses

Results are given as the mean ± standard error of the mean (SEM). Statistical analyses were performed with Excel (Microsoft Corp.) applying the 2-tailed t test. Differences of means were considered significant at a significance level of 0.05 (n.s, not significant; *P* > 0.05; *, *P* ≤ 0.05); **, *P* ≤ 0.01).
